# A Functional Role for the Monomethylated Gln-51 and Lys-53 Residues of the 49GGQTK53 Motif of eL42 from Human 80S Ribosomes

**DOI:** 10.2174/1874091X01711010008

**Published:** 2017-03-31

**Authors:** Stéphanie Eustache, Jean-Bernard Créchet, Tahar Bouceba, Jun-ichi Nakayama, Mayo Tanaka, Mieko Suzuki, Anne Woisard, Pierre Tuffery, Soria Baouz, Codjo Hountondji

**Affiliations:** 1 Sorbonne Universités, UPMC Univ Paris 06, Laboratoire “Enzymologie de l’ARN”, UPMC-UR6, (Tour 32), Case courrier 60 - 4, Place Jussieu, F-75252, Paris Cedex 05, France; 2 Université Paris-Diderot, Sorbonne-Paris-Cité, INSERM-UMR-S973 and RPBS, Paris, France; 3 Ecole Polytechnique, Route de Saclay, F-91120 Palaiseau, France; 4 Sorbonne Universités, UPMC Univ Paris 06, Institut de Biologie Paris Seine (IBPS) Plateforme d’interactions moléculaires, CNRS-FR3631; 7, Quai Saint Bernard, F-75252, Paris Cedex 05, France; 5 Graduate School of Natural Sciences, Nagoya City University, 1 Yamanohata, Mizuho, Nagoya, Aichi 467-8501 Japan

**Keywords:** eL42 protein, Human 80S ribosomes/GGQTK motif, eL42/monomethylated Gln-51, Lys-53 residues, eL42/human translation termination factor, eRF1/tRNA, 28S rRNA/binding assays, Biacore

## Abstract

**Background:**

We have previously demonstrated that the eukaryote-specific ribosomal protein eL42 of the human 80S ribosome contains seven monomethylated residues, among which are the Gln-51 and Lys-53 residues contained in the 47GFGGQTK53 sequence conserved in all eukaryotic 80S ribosomes. This sequence contains the methylated and universally conserved GGQ motif common for all class-1 translation termination factors responsible for stop codon recognition and for triggering the hydrolysis of the P site-bound peptidyl-tRNA. We have also recently reported a model of ribosomal ternary eL42-tRNA-eRF1 complex where specific regions of all three macromolecules (the comparably flexible GGQ domains of eRF1 and eL42 and the CCA-arm of tRNA) are involved in interactions.

**Method:**

Here, we have studied the interactions between recombinant eL42 and eRF1 proteins and the tRNA substrate by means of the Biacore assay, using the wild-type eL42 protein, the eL42-Δ(GGQTK) mutant (the eL42 protein whose GGQTK motif has been deleted), the single Q51E and K53Q mutants (eL42-Q51E and eL42-K53Q, respectively), as well as the double Q51A/K53A mutant (eL42-Q51A/K53A).

**Results:**

Our results show that the monomethylated Gln-51 and Lys-53 residues contained in the 47GFGGQTK53 sequence of eL42 and the monomethylated GGQ motif of eRF1 represents the sites of interaction between these two proteins through hydrophobic contacts between methyl groups. We also demonstrate that the interactions between eL42 and tRNA or 28S rRNA are characterized by strong binding affinities (*K*_D_ values in the nanomolar or picomolar range, respectively) which argue for specific interactions. Strong interactions between eL42 and tRNA are likely to be responsible for the decrease in the poly(U)-dependent poly(Phe) synthesis activity of human 80S or *E. coli* 70S ribosomes in the presence of added human recombinant eL42. It is proposed that the decrease of the activity of the ribosome is caused by the sequestration of the substrate Phe-tRNA^Phe^ by the added eL42 protein.

**Conclusion:**

Interactions between the monomethylated Gln-51 and Lys-53 residues of the 49GGQTK53 motif of the human eL42 protein and the methylated GGQ motif of eRF1 are likely to play a functional role on translating human 80S ribosomes.

## INTRODUCTION

Protein methylation, the transfer of a methyl group to amino acids, such as lysines, arginines and histidines, is an essential process for the regulation of gene expression, protein function, and RNA metabolism [1, 2]. Historically, histones have been the most extensively studied among the many substrates for protein methylation. Indeed, methylation of histones, in combination with other modifications, constitutes the ‘histone code’ that determine chromatin structure and DNA accessibility for replication, repair, and transcription [3]. Other substrates for methylation include components of the translational apparatus. In fact, in addition to methylated rRNAs and tRNAs, several ribosomal proteins (rps) and translation factors (TFs) are also methylated [4]. Particularly, methylation of rps may play crucial roles in RNA binding and/or in protein–protein interactions within the ribosome [5]. An example of protein-RNA interaction is the involvement of Arg methylation in the formation and stability of ribosome-mRNA complexes [6]. As for the protein–protein interactions within the ribosome, they are examplified by the formation of the bL12 stalk (formerly L7/L12 stalk) of bacterial ribosomes, a pentamer of two bL12 (formerly L7/L12) and one bL10 (formerly L10) which is involved in the interactions with TFs. In the latter case, the monomethylated Lys-81 appears to be at the interface of the two bL12 molecules which form each of the two dimers. Monomethylation at Lys-81 is likely to participate to the dimer formation or binding to other RPS [7, 8]. In this context, our recent studies have shown that the eukaryote-specific large subunit ribosomal protein eL42 of the human 80S ribosome contains seven monomethylated residues, among which are the Gln-51 and Lys-53 residues contained in the 47GFGGQTK53 sequence conserved in all eukaryotic 80S ribosomes [9]. This sequence contains the methylated and universally conserved GGQ motif common for all class-1 translation termination factors responsible for stop codon recognition and for triggering the hydrolysis of the P site-bound peptidyl-tRNA [10-13]. Several research groups have demonstrated that the GGQ motif of the translation termination factors is in direct contact with the peptidyl transferase center (PTC), the active site of the ribosome located on the large subunit [10, 12, 14-18]. However, the PTC is still not defined in molecular terms. Interestingly, we have demonstrated recently that the Gln-53 and Lys-55 residues of the 49GFGGQTK55 motif of eL42 from *Schizosaccharomyces pombe* (*S. Pombe*) contribute in some active and direct way to catalysis of peptide bond formation (unpublished results). According to these data, one possible role of the GGQ minidomains of eL42 and of the translation termination factor eRF1 might consist in interacting with each other and with the CCA-arm of P-tRNA. This possibility prompted us to study the interactions between the recombinant wild-type or appropriate mutant eL42 proteins and eRF1 or the tRNA substrate by means of the Biacore assay. Our results show that the methylated residues of the GGQ minidomains of the human proteins eL42 and eRF1 represent the sites of interaction between these two proteins through hydrophobic contacts between methyl groups.

## MATERIALS AND METHODS

### Translation Factors and tRNA

Purified elongation factors from calf brain EF-1α, EF-1β, EF-2 were obtained as described in [19]. tRNA^Asp^ was purified as previously described [20]. Poly(U) and tRNA^Phe^ from *E.coli* were from Sigma-Aldrich and L-[^14^C(U)]Phenylalanine (18GBq.mmol^-1^).

tRNA^Phe^ was aminoacylated using [^14^C(U)] Phenylalanine with an excess amount of partially purified phenylalanyl-tRNA synthetase from *E. coli* as in [21, 22]. Human eRF1 was a gift from Heurgué and Mora.

#### Purification of EF-3

TKBL40 (BL21 Lex His-Tagged eEF-3 pET IIa was a gift of Maria Mateyak (Rutgers RWJ MED School, Biochemistry and molecular Biology department, Piscataway, US). A fresh overnight saturated culture was used to inoculate 2 liters of LB with 50 µg.ml^-1^ ampicillin and grown with shaking at 37°C to 0.6 A_600_, after which induction with 0.2 mM isopropyl-ß-D-thiogalacto-pyranoside took place at 23°C for 15 hours. After harvest, the cells (14g) were washed in PBS, sonicated 15 times for 10 secondes at 4°C in 40 ml buffer B (25 mM Tris-HCl pH 7.5, 0.5M NaCl, 10 mM Imidazole, 1 mM ß-ME) containing, 1 mM MgCl_2_, 0.5 mg ml^-1^ lysosyme, 100 µg ml^-1^ DNase, one tablet of complete mini EDTA free protease inhibitor cocktail (Roche Diagnostics), and centrifuged (120,000 g for 30 minutes). The supernatant was applied two times on 1 ml His graviTrap column (GE Healthcare) equilibrated in buffer B. After washing with 20 ml buffer A containing 20 mM imidazole, EF-3 was eluted in 10 ml buffer B with 100 mM imidazole. This fraction was concentrated in an Amicon ultrafiltration apparatus (Millipore membrane PM10), dialysed against 25 mM Tris-HCl pH7.5, 1 mM MgCl_2_, 50mM NH_4_Cl, 1 mM dithiotreitol, 50% glycerol and stored at -30°C.

#### Purification of the Human Recombinant eL42-∆(GGQTK) Variant

A 5’ coding region of human eL42 (formerly RPL36A) was PCR amplified from pColdI-RPL36A [23] using a 5’ primer GCTCGGTACCCTCGAGGGATCC of pColdI, and a 3’primer CCTTTTTCCGGAAAATCGGATAGCCACT CTGCTTCC corresponding to a region of eL42 deleted of residues 49-GGQTK-53 respectively containing the *Xho*I and *Bsp*EI restriction sites (underlined). The digested *Xho*I–*Bsp*EI purified PCR fragment was then cloned in the corresponding sites of pColdI-RPL36A as a replacement of the wild type sequence leading to pcoldI-RPL36A(∆ GGQTK). Accuracy of the amplification was controlled by sequencing of the cloned gene. Competent Solu BL21 *E. coli* cells (AMS Biotechnology) were transformed with pcoldI-RPL36A(∆ GGQTK). His-tagged eL42-∆(GGQTK) was then expressed and purified as described for the purification of wild type eL42 [23].

#### Construction and Purification of the eL42-Q51E, eL42-K53Q and eL42-Q51A/K53A Mutants

Mutations were introduced with the QuickChange II XL Site-Directed Mutagenesis Kit and by using the manufacturer (Stratagen) recommended protocol. As a substrate for the mutagenesis reactions we used pColdIRPL36A plasmid. The mutagenic oligonucleotides containing the specific modified bases and their respective complementary primers for introducing the required amino acid substitutions on eL42 were synthetized and purified by Eurogentec.

Following transformation of competent XL10-Gold cells, minipreps on selected transformants were analyzed by sequencing to verify the presence of desired mutations and to check the absence of secondary mutations.

Competent Solu BL21 *E. coli* cells were transformed with the respective pcoldI-RPL36A mutated plasmids and expression and purification were performed as desribed for the purification of wild type eL42.

#### Construction of DNA Template and Synthesis of the 28S rRNA Fragment

A fragment of human 28S rRNA (246 nt long) containing sequences 3898-3937/4173-4234/4327-4405/ 4438-4458/4520-4563 under control of T7 promoter was produced by *in vitro* transcription utilizing the T7 RNA polymerase [24]. A DNA template for the synthesis was constructed using long PCR-based fusion technique [25]. In the first step, a plasmid pHr13 [26] containing human 28S rRNA gene was used as a template in amplifications with the following pairs of primers: forward primer (primer A) 5’-GGATCCTAATACGACTCACTATAG GGAAAGAAGACCCTGTT-3’, reverse primer 5’-GTGCCAGACTAGAGTCAAGC-3’; forward primer 5’-GCTTGA CTCTAGTCTGGCACGTGCCAGGTGGGGAGTTTGA-3’, reverse primer 5’-TTAGGACACCTGCGTTACCG-3’; forward primer 5’-GGTAACGCAGGTGTCCTAACGGGGCCTCACGATCCTTCTG-3’, reverse primer 5’-CAGGCCAGTTATCCCTGTGG-3’; and forward primer 5’-GGGATAACTGGCCTGTTGATCCTTCGATGTCGGCT CGTGAGCTGGGTTTAG-3’ reverse primer (primer B) 5’-CTGCAAGGGTAAAACTAACCTGTCTC-3’.

The products of these PCR amplifications were used as templates in the second PCR, which was performed with primer A (forward) and primer B (reverse). The PCR product was cloned in pUC19 vector digested with *Sma*I. The integrity of the resulting insertion was verified by sequencing.

#### Poly(U)-Dependent Poly(Phe) Synthesis Activity

Poly(Phe) synthesis was determined as incorporation of L-[^14^C(U)]Phenylalanine into hot trichloroacetic acid-insoluble material as described in [19]. The reaction mixture (100 µl) contained 40 mM Tris-HCl pH 7.5, 7 mM MgCl_2_, 80 mM NH_4_Cl, 1 mM dithiothreitol, 1 mM ATP, 1 mM Phospho*enol*pyruvate, 0.3 mM creatine phosphate, 0.5 mM GTP, 50 µg.ml^-1^ pyruvate Kinase, 50 µg.ml^-1^ creatine Kinase 5 µM tRNA^Phe^ (first charged during a 30 minutes incubation at 30°C with a 2 fold excess of L-[^14^C(U)]Phenylalanine (5 GBq.mmol^-1^)) and a saturating amount of partially purified phenylalanyl-tRNA synthetase), 3.5 µg poly(U) and 0.5 µM EF-1α, 0.15 µM EF-1β, 0.35 µM EF-2, and 14 pmol of human 80S ribosome. In the prokaryotic poly(U)-dependent poly(Phe) synthesis assay, 0.5 µM EF-Tu, 0.2 µM EF-Ts and 0.2 µM EF-G were used, and the reaction was started with 0.35 µM of 70S ribosomes. During incubation at 30°C, 30 µl aliquots were withdrawn at times indicated, spotted on glass fiber filters and hot trichoroacetic acid insoluble radioactivity was determined.

#### Surface Plasmon Resonance

To obtain quantitative kinetic measurements of the interactions between the eL42 protein and various partners, experiments were conducted on a Biacore 3000 instrument (GE Healthcare) of the Platform of Molecular Interactions of the IBPS Institute (Université Pierre et Marie Curie). All experiments were performed in triplicate, as described previously [23]. The interaction was analyzed between two partners: one partner immobilized on the sensor chip was called ligand. The second partner, passed in a continuous flow over the ligand surface is called analyte. Two different sensor chips were used. A NTA sensor chip for His-tagged ligand immobilization, and a CM5 sensor chip, as an alternative to NTA when His-tagged analyte was used. SPR experiments on CM5 sensor chip: purified His-tagged eL42 was immobilized through primary amino groups to the carboxy-methylated dextran matrix of a CM5 sensor chip. A solution of 1 μM His-tagged eL42 diluted in immobilization buffer (10 mM sodium acetate, pH 5.5) was injected at a flow rate of 10 μL/min during 7 min, in order to obtain 3,900 RU of covalently coupled protein. The immobilization was followed by injection of 70 μL 1 M ethanolamine hydrochloride, pH 8.5, at a flow rate of 10 μL/min to saturate the free activated sites of the carboxy-methylated dextran matrix. A reference surface without protein was prepared using the same procedure. Kinetic experiments were carried out at 25°C at a flow rate of 5 μL/min. Buffer HBSEP (10 mM HEPES, pH 7.4, 150 mM NaCl, 3 mM EDTA, 0.005% surfactant P20) was used as the running buffer. Sensorgrams were obtained by passing various concentrations of the analyte over the ligand surface at a flow rate of 5 μL/min, with a 5-min association phase and a 8-min dissociation phase. The sensor surface was regenerated between each cycle (association-dissociation) with one injection of 10 mM glycine hydrochloride (pH 2.0) at a flow rate of 30 μL/min during 30 sec. When necessary, the regeneration was completed by injection of 1 M NaCl and 30 mM NaOH in the same conditions. Identical injections over blank surfaces run in parallel (and giving a value of 0 RU) were subtracted from all experiments. SPR experiments on NTA sensor chip: the purified His-tagged eL42 protein was immobilized on NTA sensor chip (GE Healthcare) to reach an immobilization level of the protein of 5,000-7,000 resonance units (RU), according to the manufacturer’s recommendations. HBS-P (10 mM HEPES pH 7.4, 150 mM NaCl, 0.005% surfactant P20) was used as running buffer. Evaluation of non-specific background signals was performed in parallel by passing analyte on NTA chips uncoated with ligand. Between the injections, the surfaces were regenerated by injection of 5 mM NaOH in the same conditions as the regeneration of the CM5 sensor chip. Kinetics were evaluated by using the BIAevaluation software, Version 4.1 (GE Healthcare). The data were processed by fitting the binding profiles to a 1:1 Langmuir interaction model. The quality of the fit was assessed by the statistical chi2 value provided by the software (chi2 values < 10 were considered as acceptable). The fitting of each dataset yielded rates for association (*k*a or *k*on) and dissociation (*k*d or *k*off), from which the equilibrium dissociation constant *K*_D_ was calculated (*K*_D_ = *k*off /*k*on). The *k*on, *k*off and *K*_D_ from 3 experiments were used to calculate the mean values of these variables.

## RESULTS

### The Recombinant eL42 Species Under Study

The recombinant human eL42 species studied in the present report are: the wild-type eL42 protein, the eL42-Δ(GGQTK) mutant (i.e. the eL42 protein whose GGQTK motif has been deleted), the single Q51E and K53Q mutants (named eL42-Q51E and eL42-K53Q, respectively), as well as the double Q51A/K53A mutant named eL42-Q51A/K53A. A prerequisite for the study of the interactions between the recombinant eL42 species used and their partners is to demonstrate that these mutant eL42 proteins are folded similarly to the wild-type protein. To this end, we have compared the CD spectra of the wild-type eL42 protein with that of the aforementioned mutant proteins. As shown in Fig. (**1**), the CD spectra of the eL42 mutant species used in this study were not superimposable on that of the wild-type eL42. For example, the spectrum of the wild-type protein showed two negative bands at 190 and 195 nm, whereas that of the deletion mutant had only a single negative band at 196-197 nm which was stronger than those found in the wild-type’s spectrum. In addition, the spectra of the substitution mutants exhibited no distinct negative band at the region of 190-200 nm. These results suggest that the secondary and tertiary structures of the eL42 mutants are different at least in part from those of the wild-type protein. As a control, the CD spectrum of the helix-rich *E. coli* large subunit ribosomal protein bL12 is also shown Fig. (**1**). The α-helix conformation of the latter protein is confirmed by the presence of two negative peaks at 208 and 222 nm Fig. (**1**). Finally, it was verified that the wild-type eL42 protein used in this study contains the same post-translational modifications as those identified previously on the endogenous large subunit ribosomal eL42 protein ([9] and results not shown). Next, we performed binding assays on Biacore by using these purified recombinant human eL42 species and their partners such as tRNA, rRNA and eRF1.

#### Interaction Between the eL42 Species and eRF1

Surface plasmon resonance (SPR) analyses with a Biacore biosensor were carried out to determine the binding affinities of the eL42 species to eRF1, tRNA or rRNA. The wild-type eL42 protein and the eL42-Δ(GGQTK), eL42-Q51E, eL42-K53Q and eL42-Q51A/K53A mutants were immobilized each on a CM5 sensor chip for the experiments regarding their interaction with other His-tagged proteins such as eRF1. Fig. (**2A**) shows a kinetic measurement of human eL42:eRF1 interaction. The corresponding kinetic and affinity constants, as deduced from Fig. (**2A**) are: association (*k*a or *k*on) and dissociation (*k*d or *k*off) rates of 5.3 x 10^+3^ M^-1^.s^-1^ and 1.99 x 10^-4^ s^-1^, respectively, resulting in a binding affinity (*K*_D_) of 3.75 x 10^-8^ M (Table **1**). This *K*_D_ value would reflect a good binding affinity between eL42 and eRF1. By contrast, when the same experiment was conducted with eL42-Δ(GGQTK), the human mutant eL42 whose GGQTK motif has been deleted Fig. (**2B**), the kinetic and affinity constants, were: *k*on and *k*off of 1.56 x 10^+2^ M^-1^.s^-1^ and 4.87 x 10^-4^ s^-1^, respectively, resulting in a binding affinity (*K*_D_) of 3.12 x 10^-6^ M (Table **1**). The fact that the *K*_D_ value of binding to human eRF1 of the human mutant eL42 lacking the GGQTK motif is higher by two orders of magnitude than that of the wild-type eL42 would suggest that the methylated GGQTK motif plays a role in positioning key residues for the interaction with eRF1. To identify the amino acid residue(s) of the methylated GGQTK motif of eL42 involved in the interaction with eRF1, different mutants of human eL42 were constructed with the help of site directed mutagenesis by changing Gln-51 and/or Lys-53 by Glu and Gln, respectively. Thus, the eL42 mutant proteins constructed contained the single Q51E or K53Q or the double Q51A/K53A substitutions. Figs. (**2C**, **2D** and **2E**) show kinetic measurements of the interaction between eRF1 and the eL42-Q51E, eL42-K53Q and eL42-Q51A/K53A mutants, respectively. As shown in Table **1**, the *K*_D_ values of binding of eRF1 to the eL42-Q51E, eL42-K53Q and eL42-Q51A/K53A mutants were found equal to 1.92 x 10^-6^ M, 1.28 x 10^-6^ M and 1.62 x 10^-6^ M, respectively. Thus, the binding affinities of the eL42-Δ(GGQTK) and Q51A/K53A mutants to eRF1 were comparable, as expected, suggesting that both Gln-51 and Lys-53 residues might contribute to the binding of eL42 on eRF1. Surprisingly, the binding affinities of the eL42-Q51E and eL42-K53Q mutants were also found comparable with that of the eL42-Δ(GGQTK) or the eL42-(Q51A/K53A) mutants. At this stage, to rationalize the data obtained with the Biacore analyses, we have compared the position of the methylated GGQ motif in the 3-D structure of human eRF1 with that of the human eL42 protein. On one hand, we had previously modeled the human eL42 protein [23] into the structure of the archaeal ortholog extracted from the known X-ray structure of the 50S subunit of *Haloarcula marismortui* [27]. On the other hand, the crystal structure of human eRF1 is organized into three domains arranged into an overall Y-like shape [14]. The N domain contains the NIKS loop, thought to interact with the stop codon, the M domain contains the universally conserved catalytic GGQ motif located at an exposed tip of this domain, and the C domain contains residues critical for interaction with eRF3 [12, 14]. The distance between the GGQ motif at the tip of domain M and the NIKS loop responsible for codon interaction at the tip of domain N is compatible with human eRF1 being a tRNA mimic, with the N, M and C domains of eRF1 corresponding to the anticodon loop, aminoacyl acceptor stem, and T stem of a tRNA molecule, respectively [12, 14]. As illustrated in Fig. (**3**), comparison of the rp eL42 3-D structure [23] with that of the M domain of eRF1 revealed that the secondary structure elements that make up their overall structure are different. However, they present the same overall shape and size, with the GGQ loop of human eL42 positioned similarly to the corresponding loop of human eRF1 at one extremity Fig. (**3**). Moreover, both GGQ motifs are in proximity of binding in these 3-D structures. This situation is not contradictory, since the tertiary structures of bacterial and eukaryotic RFs are quite distinct [12]. For example, the tertiary structure of human eRF1 [14] shares no similarity with that of *E. coli* RF2 [13, 28]. None of the three domains of the former resembles any domain in the latter [13, 28], indicating that, despite their functional similarity, human eRF1 and *E. coli* RF2 are completely distinct proteins in terms of secondary and tertiary structures. Therefore, the structural similarities between human eL42 and eRF1 as shown in Figs. (**3A** and **3B**) are likely to reflect evolutionary conservation of the GGQ minidomains of these proteins.

#### Interaction Between the Human eL42 and the Yeast EF-3 proteins In Vitro

It has been previously reported that the elongation factor 3 (EF-3) from *S. cerevisiae* is required for the poly(U)-dependent poly(Phe) synthesis activity of the yeast 80S ribosomes [29, 30]. Moreover, it was previously demonstrated that the primary ribosome binding domain of EF-3 is located in the carboxyl-terminal end of the protein, which contains blocks of lysine residues [30]. This domain contains also a glycine-rich 896GLSGGQ901 peptide [30]. The presence of a GGQ motif in the primary ribosome binding domain of EF-3 suggests that this motif might be involved in the interaction with a ribosomal component. Since the eL42 proteins from human and yeast were shown to share 82% primary structure similarities, the GGQ domain common to these two rps is likely to represent the binding site for EF-3, even though this elongation factor is dispensable for the activity of human ribosomes. To address the question of the interaction between EF-3 and eL42, we have analyzed the kinetic of the interaction between EF-3 and the human wild-type eL42 or the eL42-Δ(GGQTK) mutant Figs. (**4A**, **4B**). As shown in Table **1**, the *K*_D_ values of binding of EF-3 to the wild-type eL42 or the eL42-Δ(GGQTK) mutant protein were found equal to 1.51x10^-7^ M and 0.92x10^-5^ M, respectively. These results suggest that the GGQTK motif of eL42 is important to define the binding site of the GGQ domain of EF-3 on the 80S ribosome. Control experiments to assess the specificity of the protein:protein interactions studied in the present report consisted in analyzing (i) the binding on eL42 of anti eL42 antibodies Fig. (**5A**) and (ii) the interactions between the eL42 protein and another RNA-binding protein that does not interact with the ribosome. In the latter case, we have used tRNA nucleotidyl transferase, the enzyme which is in charge of repairing the CCA-arm of tRNA. As shown in Figs. (**5B**, **5C** and **5D**), tRNA nucleotidyl transferase referred to as CCAse is not capable of binding to eL42 or to the eL42-Δ(GGQTK) and Q51A/K53A mutants. The fact that CCAse is not capable of binding to either eL42 species indicates that these proteins do not bear on their surface any site recognized by this tRNA repair enzyme Figs. (**5B**, **5C** and **5D**). By contrast, as expected, anti eL42 antibodies were shown to specifically recognize eL42 whatever the sensor chip (NTA or CM5) used for protein immobilization, while the anti eRF1 antibodies did not Fig. (**5A**).

#### Interaction between eL42 and RNA

It is generally accepted that methylation of rps may also play crucial roles in RNA binding. To check this hypothesis, we have analyzed the kinetic of the interaction between tRNA or rRNA and the human wild-type eL42 or the protein with six amino acids deletion, eL42-Δ(GGQTK).

To this end, each purified His-tagged protein was immobilized on NTA sensor chip. Experimental data from individual kinetic binding experiments with tRNA were analyzed and fitted using BIAevaluation software with 1:1 binding model, in accordance to the 1:1 stoichiometries of crosslinking of the recombinant or the endogenous eL42 protein with tRNAox [23]. The kinetic constants obtained are given in Table **1**. It is interesting to note that the affinities of tRNA or 28S rRNA binding to the mutant eL42 protein lacking the GGQTK motif are of the same order of magnitude than that of the wild-type eL42 Figs. (**6A**, **6B**, **6C** and **6D** and Table **1**), suggesting that the GGQTK region is not involved in specific interactions with RNA. Finally, as shown in Figs. (**6E**, **6F**) no interaction took place between eRF1 and tRNA or 28S rRNA, while both nucleic acids were shown to interact with both eL42 species.

#### Effects of added recombinant eL42 protein to the activity of human 80S ribosomes

Another question that we have addressed in the present report is the effect of added eL42 protein on the activity of human 80S ribosomes. This question stemmed from the following observations: (i) with the combination of biochemical and genetical approaches, we have recently demonstrated that the ribosomal protein eL42 from eukaryotes is indispensable for the activity of the yeast 80S ribosomes (unpublished data); (ii) the human eL42 protein was previously shown to be overexpressed in human hepatocellular carcinoma (HCC) as well as in several human tumor cell-lines [31, 32]. The authors have concluded that eL42 plays a role in tumor cell proliferation and may be a potential target for anticancer therapy [32]. For example, one explanation of the overexpression of eL42 in cancer cells is that this rP essential for the elongation step of protein biosynthesis could be overproduced to enhance the rate of the translation process in order to sustain their capacity of hyperproliferation. We addressed this question by measuring the poly(U)-dependent poly(Phe) synthesis activity of the human 80S ribosomes in the presence of added human recombinant eL42 protein. As shown in Fig. (**7A**), not only the activity of the human 80S ribosomes is not increased in the presence of added eL42 protein, but it was decreased as a function of increasing concentrations of the protein. A similar effect was observed with *E. coli* 70S ribosomes Fig. (**7B**), suggesting that eL42 interferes with one of the components of the translation apparatus. The fact that binding assays on Biacore using eL42 and tRNA had revealed that the interactions between these macromolecules are characterized by strong binding affinities (in the nanomolar range) (Table **1** and [23]) tempted us to speculate that the decrease in the poly(U)-dependent poly(Phe) synthesis activity in the presence of added eL42 might reflect the sequestration of the substrate [^14^C]Phe-tRNA^Phe^ that would prevent incorporation of [^14^C]Phe into the trichloroacetic acid-insoluble poly([^14^C]Phe) chain. As shown in Fig. (**7C**), the latter hypothesis is consistent with the observation that, when [^14^C]Phe incorporation has reached a plateau value at time t, prior to the addition of eL42, no further decrease in the activity of *E. coli* 70S ribosomes was visible. The same results were obtained with the human 80S ribosomes. Finally, it was verified that the decrease in the poly(U)-dependent poly(Phe) synthesis activity of human 80S or *E. coli* 70S ribosomes as a function of increasing concentrations of the protein is the same for the wild-type eL42 or the eL42-Δ(GGQTK) mutant Fig. (**8**). This result suggests that the GGQTK motif of eL42 is not involved in specific interactions with tRNA, as discussed above.

## DISCUSSION

### Earlier Studies on eL42

Several research groups have demonstrated that the GGQ motif common to all translation termination factors is in direct contact with the PTC [10, 12, 14-17]. Since the PTC is supposed to be composed of rRNA only, it was proposed that the GGQ motif would display RNA-binding properties toward rRNA or peptidyl-tRNA. However, the previously reported model of ribosomal ternary eL42-tRNA-eRF1 complex [23] where specific regions of all partners (*i.e.* the comparably flexible GGQ domains of eRF1 and eL42, as well as the CCA-arm of P-tRNAox) are involved in a crosslinking reaction, suggests that the role of the GGQ minidomain of eL42 and eRF1 might consist in interacting with each other and with the CCA arm of P-tRNA. Therefore, in the present report, we have studied the interactions between recombinant eL42 and eRF1 proteins and the tRNA substrate by means of the Biacore assay. However, a prerequisite for this study is to demonstrate that specific interactions do exist between tRNA, eL42 and eRF1 on and off the ribosome. On one hand, we have modeled recently the 3-D structure of the human eL42 protein alone or in complex with tRNA, and the models obtained were shown to exhibit specific interactions reminiscent of the general model of tRNA-aminoacyl-tRNA synthetase interaction [23]. Moreover, previous binding assays on Biacore using purified recombinant human eL42 protein and tRNA have revealed that these interactions are characterized by strong binding affinities [23]. Finally, crosslinking studies on the endogenous eL42 protein *in situ* on human 80S ribosomes [9], as well as on the human recombinant eL42 [23] have shown that this large subunit ribosomal protein is crosslinked similarly with tRNAox on and off the ribosome [23]. Altogether, the previously reported data cited above had led to the conclusion that the human eL42 protein is so far the only ribosomal protein that interacts with tRNA on and off the ribosome [23]. The aforementioned crosslinking studies have shown that the lysyl residue 53 of eL42 is the site crosslinked *in situ* with tRNAox on the human 80S ribosomes [9]. Recently, we have demonstrated that the pKa value of the ε-amino group of Lys-53 (pKa = 6.9 + 0.1) is the same on the recombinant human eL42 (unpublished data) and on the endogenous eL42 protein crosslinked with tRNAox [23]. These results suggest that other ribosomal components do not influence significantly the reactivity of Lys-53 on the human 80S ribosome. On the other hand, when human or yeast eRF1 were incubated with tRNAox in the buffer conditions that we routinely use for the crosslinking reaction with the aminoacyl-tRNA synthetases [33-36], a covalent 1:1 eRF1-tRNAox complex was formed (results not shown), suggesting that, similarly to eL42, eRF1 is capable of contacting the CCA-end of tRNA, as expected. Taking into account all these data, we assume that, if similarly to the general model of tRNA-aminoacyl-tRNA synthetase interaction [34-36], the tRNA substrate is capable of interacting with eL42 [23] and with eRF1 off the ribosome, then it would be capable of interacting with these proteins on the ribosome as well. Therefore, the interactions between recombinant human eL42 protein and its ribosomal partners such as tRNA, rRNA and eRF1, as studied by the binding assays on Biacore in the present report, are likely to reflect the interactions between these macromolecules on the human 80S ribosome.

#### The Recombinant eL42 Species Under Study

Even though the CD spectra of the eL42 mutant species used in this study were not superimposable on that of the wild-type eL42, the difference between the spectrum of the wild-type protein and that of the deletion mutant was not important. Therefore, their content of secondary structure elements and their overall 3-D structures should be roughly comparable.

The latter observation agrees well with the fact that the GGQTK motif is situated apart from the few secondary structure elements of the protein, in the extension loop at one extremity of the three dimensional structure [37]. In addition, it was verified that the wild-type eL42 protein contains the same post-translational modifications as those identified previously on the endogenous large subunit ribosomal eL42 protein. Moreover, it is well known that the human eRF1 used in the present study is methylated on the glutaminyl residue 185 as are all eukaryotic and prokaryotic release factors.

#### Interactions Between the eL42 Protein and Translation Factors

The *K*_D_ values of binding to eRF1 of the eL42-Δ(GGQTK) and eL42-Q51A/K53A mutants were found to be higher by two orders of magnitude than that of the wild-type eL42, suggesting that the binding affinities of eRF1 to these eL42 mutants are lower than to the wild-type eL42. A straightforward interpretation of these results is that the GGQTK motif of eL42 is in direct contact with that of eRF1. Therefore, the fact that Gln-51 and Lys-53 of the 47GYGGQTK53 **heptamer sequence** of the human eL42 protein were previously shown to be methylated to about 50% [9], while the human eRF1 is well-known to be methylated on the glutaminyl residue of the 183GGQ185 motif, as are all eukaryotic and prokaryotic release factors, makes it tempting to propose that the methylated residues of the wild-type eL42 protein and of eRF1 represent sites of interaction between these two proteins through hydrophobic contacts between methyl groups. However, the reason why the *K*_D_ values and the binding affinities of the eL42-Q51E and eL42-K53Q mutants are comparable with that of the eL42-Δ(GGQTK) and eL42-Q51A/K53A mutants is unknown. One possible explanation is that the interaction between eL42 and eRF1 through their methylated GGQ regions is accompanied by conformational changes in the 3-D structure of either partner that might result in a synergistical effect strengthening the interaction. Similarly, the *K*_D_ value of binding to EF-3 of the eL42-Δ(GGQTK) mutant was found to be higher by two orders of magnitude than that of the wild-type eL42, suggesting that the 47GYGGQTK53 motif of eL42 might define the binding site of the GGQ domain of EF-3 on the 80S ribosome. As shown in Table **1**, the decrease of the binding affinities of the translation factors for all eL42 mutant proteins is correlated with the decrease of their association rates with regard to that of the wild-type eL42. Also, the number of complexes formed at equilibrium between the eL42-Δ(GGQTK) and eL42-Q51A/K53A mutants and eRF1 or EF-3 was lesser than that with the wild-type eL42 Figs. (**5B**, **5C** and **5D**). Altogether, these data indicate that the association step in the binding of eRF1 or EF-3 to the eL42 mutants is severely affected, in comparison with the wild-type eL42.

#### Interactions Between the eL42 Protein and RNA

As mentioned above, the eL42:tRNA complex that we have modeled recently was shown to be analogous to the general model of tRNA-aminoacyl-tRNA synthetase interaction [23]. The latter interaction has proven to be specific owing to the large size of the aminoacyl-tRNA synthetases and of their tRNA substrates [33-36]. For example, taking into account the location of the KMSKS motif in the Rossman Fold of the class 1 aminoacyl-tRNA synthetases [38], on one hand, and the size of the tRNA molecule (75 angströms), on the other hand, it appears that only one orientation of this nucleic acid is possible upon binding to the cognate synthetase [33, 34, 38, 39]. Considering that the GFGGQTK motif of the eL42 protein binds the CCA-arm of tRNA [9], and assuming that the RNA-binding motif referred to as the nucleotide binding domain 2 (NBD2) in [23] is involved in the binding of tRNA or rRNA, it is most probable that the orientation of the tRNA molecule is unique upon binding to eL42, similarly to the model of tRNA-aminoacyl-tRNA synthetase interaction. These observations agree well with the 1:1 stoichiometry of crosslinking of the recombinant or the endogeneous eL42 proteins, as well as that of all aminoacyl-tRNA synthetases studied so far [23, 34-36, 40]. Therefore, the presence on eL42 of multiple overlapping tRNA binding sites or the binding of multiple protein molecules on one tRNA molecule could be ruled out by these arguments. In contrast to the interactions between the eL42 species and the translation factors, the affinities of tRNA or rRNA binding to the eL42-Δ(GGQTK) mutant are of the same order of magnitude than that of the wild-type eL42 (Table **1**), suggesting that the GGQ minidomain is not involved in specific interactions with RNA, as expected. In fact, as discussed above, the wild-type or the eL42-Δ(GGQTK) mutant proteins contain an RNA-binding motif [23]. This motif called nucleotide binding domain 2 (NBD2) is likely to interact with nucleotides of the putative peptidyl transferase center contained in the fragment of human 28S rRNA (246 nt long) used in the present study and/or with tRNA. Note however that, as shown in Table **1**, the association rate of tRNA binding to the wild-type eL42 protein (1.5x10^6^ M^-1^.s^-1^) is larger than to the eL42-Δ(GGQTK) mutant (5.89x10^4^ M^-1^.s^-1^). As discussed above for the protein:protein interactions between eRF1 and the eL42-Q51E and eL42-K53Q mutants, it is possible that the interaction between tRNA and other regions of eL42 such as NBD2 is accompanied by conformational changes in the 3-D structures of both macromolecules resulting in a synergistical effect in the protein:tRNA association. It should be noted that the eL42 species used in the present report are highly basic (with a pI of 10.59) so that their positive charges would strongly interact with the negative charges of RNA. These observations are consistent with the high RNA-binding affinities (*K*_D_ values in the nanomolar or picomolar range) between eL42 and tRNA or rRNA, respectively (Table **1**). The strong interactions between these macromolecules are responsible for the decrease in the poly(U)-dependent poly(Phe) synthesis activity of human 80S or *E. coli* 70S ribosomes in the presence of added eL42. They might lead to the sequestration by the added eL42 protein of the substrate [^14^C]Phe-tRNA^Phe^ that would prevent incorporation of [^14^C]Phe into the poly([^14^C]Phe) chain. It should be noted that the inhibitory effect of added eL42 suggests that cancer cells proliferation in the context of eL42 overexpression is not due neither to the enhancement of the rate of tRNA aminoacylation nor to another step of the translation process but to a still unknown mechanism. Interestingly, it is generally accepted that some overexpressed ribosomal proteins exert a direct effect on proto-oncogenes and tumorigenesis, while others interact directly or indirectly with the p53 tumor suppressor pathway. Work is in progress to determine whether the overexpression of eL42 is a causative factor of increased cell proliferation. In sharp contrast to the eL42:RNA complex, no interaction took place between eRF1 and tRNA or rRNA Figs. (**6E** and **6F**). These results might be interpreted as follows: (i) this protein is an extra-ribosomal factor which must be capable of easily dissociating from the ribosome and rebinding continuously at each termination step of the translation process, a property that is not compatible with a strong binding affinity with rRNA or tRNA; (ii) by contrast to the eL42 protein, eRF1 is an acidic protein (with a pI of 5.51) and the repulsion between its residual negative charges and those of RNA is supposed to result in a weak binding affinity. The latter argument can be applied to the interactions between tRNA and rRNA because their respective negative charges would be subject to a severe repulsion. In conclusion, the simplest reasoning about protein:RNA or RNA:RNA interactions is actually that, for a tRNA to interact with rRNA at the functional A-, P- or E-sites, its negative charges must be hidden by positive ions such as Mg^2+^ and/or by appropriate proteins.

## CONCLUSION

Mutational analysis of the monomethylated Gln-51 and Lys-53 residues of the GGQTK motif of the eL42 protein from the human 80S ribosome was performed with the goal of studying the interactions between recombinant eL42 and eRF1 proteins and the tRNA substrate by means of the binding assays on Biacore. To this end, different mutants of human eL42 were constructed with the help of site directed mutagenesis: these are the eL42-Δ(GGQTK) mutant (i.e. the wild-type eL42 protein whose GGQTK pentapeptide has been deleted), the single eL42-Q51E and eL42-K53Q mutants, as well as the double eL42-Q51A/K53A mutant. Our results show that the monomethylated Gln-51 and Lys-53 residues contained in the 47GFGGQTK53 peptide of eL42 and the monomethylated GGQ minidomain of eRF1 represent the sites of interaction between these two proteins through hydrophobic contacts between methyl groups. In addition, we demonstrate that the interactions between eL42 and tRNA or rRNA are characterized by strong binding affinities (K_D_ values in the nanomolar or picomolar range, respectively) which argue for specific interactions. We propose that strong interactions between eL42 and tRNA are responsible for the decrease in the poly(U)-dependent poly(Phe) synthesis activity of human 80S or *E. coli* 70S ribosomes in the presence of added human recombinant eL42 which might be caused by the sequestration of the substrate Phe-tRNA^Phe^ that would prevent incorporation of Phe residues into the growing poly(Phe) chain.

## Figures and Tables

**Fig. (1):**
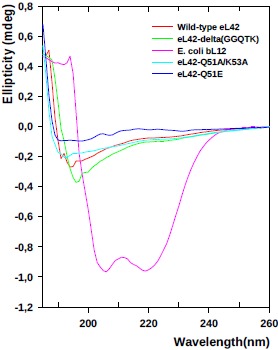
CD spectra of the eL42 species under study. CD spectra at room temperature were obtained using a Jobin Yvon spectrophotometer. Protein concentration was 29 µM except for the eL42-Q51E mutant (16.8 µM). All samples were diluted with 50 mM Tris-HCl buffer (pH 7.6). The α-helix conformation of *E. coli* bL12 is confirmed by the presence of two negative peaks at 208 and 222 nm.

**Fig. (2):**
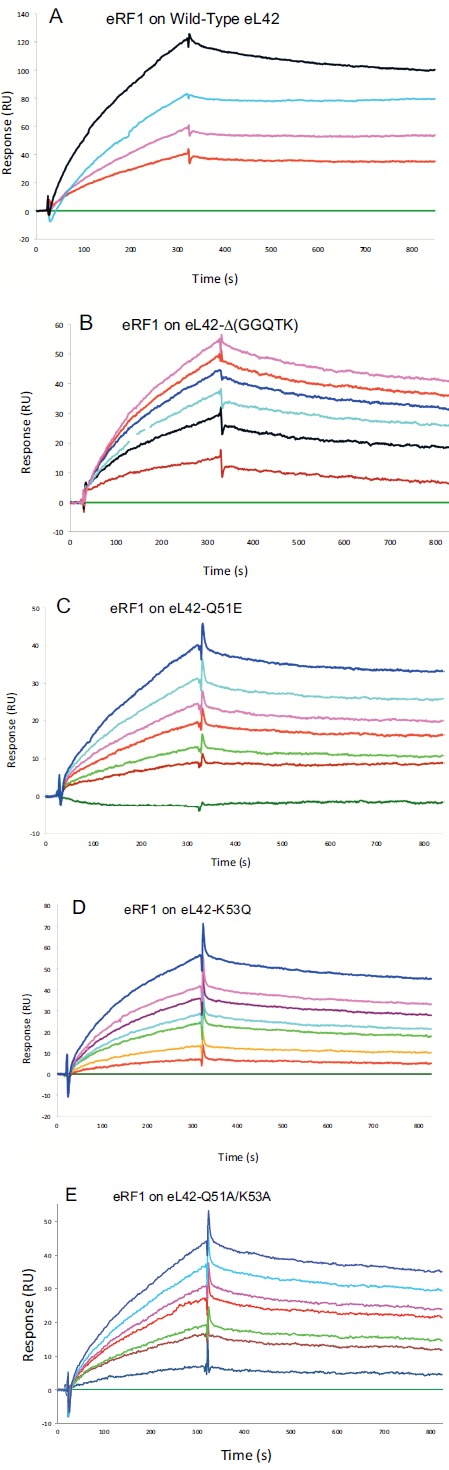
Interactions between the eL42 species and the translation termination factor eRF1. (**A**), the His-tagged recombinant human eL42 was immobilized on the surface of CM5 sensor chip at low density resonance units (RU). Various concentrations of eRF1 (0, 0.2, 0.3, 0.6 and 1 μM) were run over the chip surface. (**B**), the same experiment as in (**A**), with the His-tagged human eL42-Δ(GGQTK) mutant, the eL42 protein lacking the 49GGQTK53 pentapeptide. The concentrations of eRF1 used were: 0, 0.1, 0.2, 0.25, 0.3, 0.35 and 0.4 μM. (**C**), eL42-Q51E mutant in the presence of eRF1 at the concentrations of 0, 0.04, 0.06, 0.08, 0.1, 0.12 and 0.14 μM. (**D**), eL42-K53Q mutant in the presence of eRF1 at the concentrations of 0, 0.04, 0.08, 0.12, 0.16, 0.2, 0.24 and 0.28 μM. (**E**), eL42-Q51A/K53A mutant in the presence of eRF1 at the concentrations of 0, 0.02, 0.05, 0.06, 0.07, 0.08, 0.1 and 0.12 μM.

**Fig. (3):**
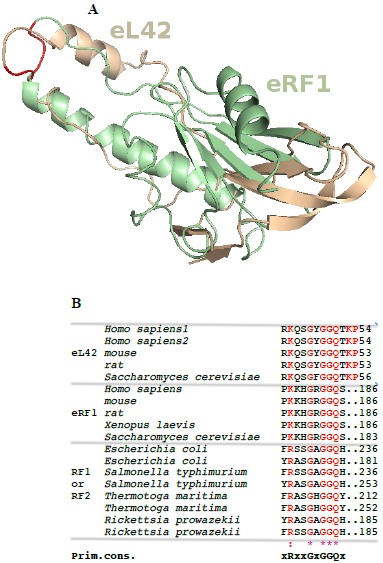
Comparison of the primary and tridimensional structures of the GGQ domain common to the eukaryotic eL42 proteins and the translation termination factors. (**A**), overlaid structures of the GGQ domain (the M domain) of human eRF1 (Acc. N. P62495) colored green and eL42 (colored brown) modeled in [23]. The methylated GGQ motif is highlightened (red). (**B**), conserved region in the GGQ domain of the eukaryotic eL42 proteins and the translation termination factors. Multiple sequence alignment of the region encompassing the GGQ motif common to the class-1 translation termination factors and the rps of the eukaryotic eL42 family was generated with the program ClustalX. The amino acid residues that are conserved in majority in either group are colored red. The bottom line labels residues as either strictly conserved (*) or highly conserved (**:**).

**Fig. (4):**
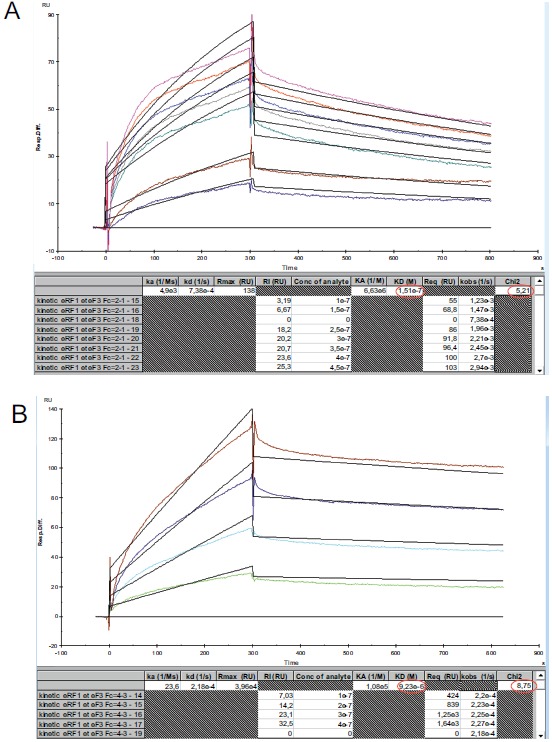
Interactions between human eL42 and the yeast elongation factor 3 (EF-3). (**A**), His-tagged human eL42 immobilized on CM5 sensor chip. Various concentrations of *S. cerevisiae* EF-3 (0, 0.1, 0.15, 0.25, 0.3, 0.35, 0.4 and 0.45 μM) were run over the chip surface. (**B**), the same experiment as in (**A**), with the His-tagged human eL42-Δ(GGQTK) mutant and with the EF-3 concentrations of 0, 0.1, 0.2, 0.3, 0.4 and 0.45 μM.

**Fig. (5):**
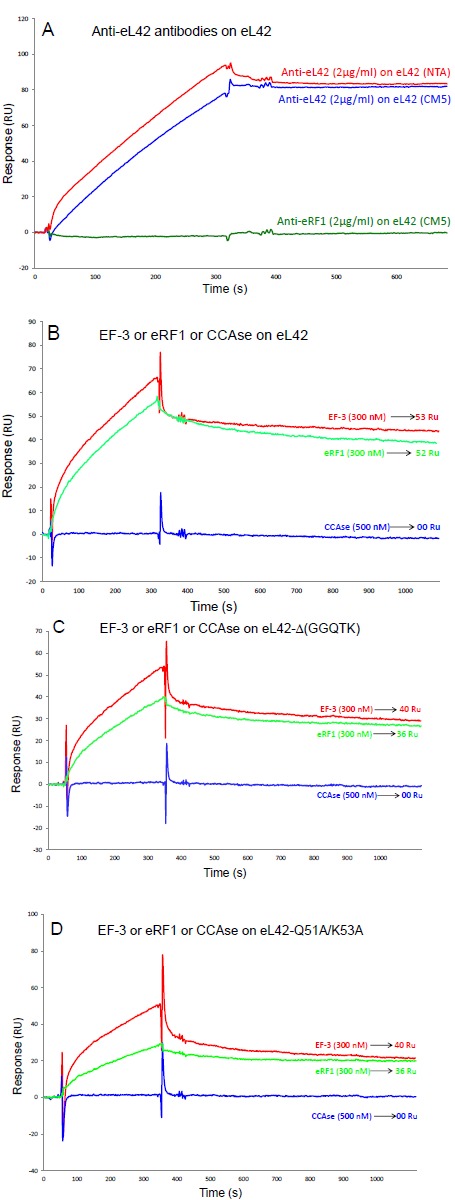
Control experiments of the interactions between eL42 and the translation factors. (**A**), positive control consisting of the measurement of the interaction between eL42 and the anti-eL42 antibodies. (**B**), negative control showing the absence of interaction between eL42 and tRNA nucleotidyl transferase (CCAse), in comparison with the translation factors eRF1 and EF-3. (**C**), the same experiment as in (**B**), with the eL42-Δ(GGQTK) variant. (**D**), negative control showing the absence of interaction between the eL42-Q51A/K53A mutant and CCAse, in comparison with the translation factors eRF1 and EF-3.

**Fig. (6):**
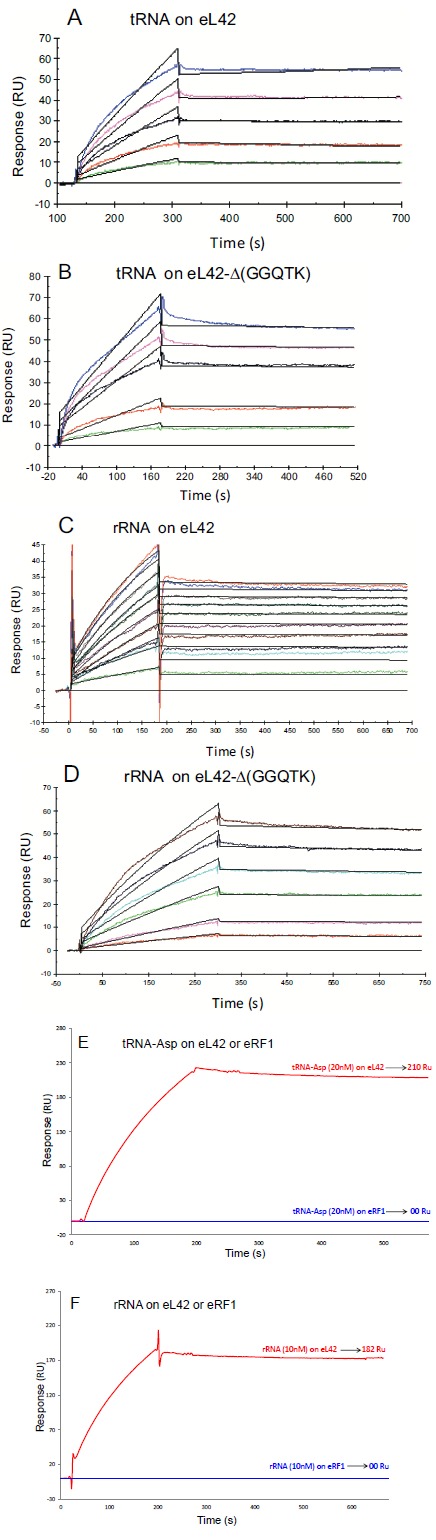
Kinetic measurement of eL42:tRNA or eL42:rRNA interactions. (**A**), His-tagged human eL42 was immobilized on the surface of NTA sensor chip at low density resonance units (RU). Various concentrations of tRNA-Asp (0, 1, 2, 4, 5 and 6 nM) were run over the chip surface. (**B**), the same experiment as in (**A**), with the His-tagged eL42-Δ(GGQTK) mutant and with tRNA-Asp concentrations of 0, 1, 2, 4, 5 and 6 nM. (**C**), interactions between His-tagged eL42 and rRNA at the concentrations of 0, 0.02, 0.040, 0.060, 0.080, 0.1, 0.12, 0.140, 0.160, 0.180 and 0.200 nM. (**D**), the same experiment as in (**C**), with the His-tagged eL42-Δ(GGQTK) mutant and with rRNA at the concentrations of 0, 0.05, 0.1, 0.2, 0.3, 0.4, and 0.5 nM. (**E**), His-tagged human eL42 and eRF1 were each immobilized on the surface of NTA sensor chip, and tRNA (20 nM) was run over the chip surface. (**F**), the same experiment as in (**E**), with rRNA (10 nM) instead of tRNA.

**Fig. (7):**
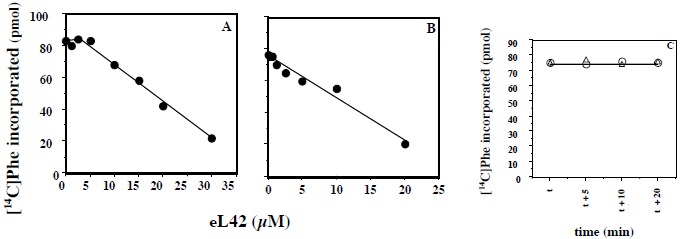
Effect of increasing concentrations of eL42 on the *in vitro* poly(U)-dependent poly(Phe) synthesis activity of (**A**) human 80S or (**B**) *E. coli* 70S ribosomes. The reaction in 30 µl was started with ribosomes after preincubation for 10 min at 30°C of increasing concentrations of eL42 with charged tRNA^Phe^. Activity was determined after 40 min incubation at 37°C in (**A**) and 10 min incubation at 30°C in (**B**). For details see Materials and Methods. (**C**): effect of eL42 addition to the incubation mixture of the poly(U)-dependent poly(Phe) synthesis reaction catalyzed by *E. coli* 70S ribosomes when [^14^C]Phe incorporation has reached a plateau value at time t, prior to the addition of eL42. After 40 min incubation at 37°C (time t), activity was determined with (∆) or without (o) addition of eL42 (20 µM final concentration) in the 70 µl reaction mixture. Activity was determined on 15 µl aliquots withdrawn at time t, t + 5 min, t +10 min, t + 20 min.

**Fig. (8):**
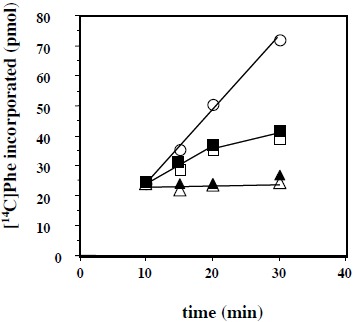
Kinetics of poly(U)-dependent poly(Phe) synthesis reaction in the absence (open circles) or in the presence of the wild-type eL42 or the eL42-Δ(GGQTK) mutant at the concentrations of 5 µM (squares) or 18 µM (triangles). For details, see Materials and Methods.

**Table 1:** Kinetic and affinity constants for the interactions between the eL42 species and tRNA, rRNA, or the translation factors eRF1 and EF-3.

Proteins	Ligands	*k*_a_ = *k*_on_ (M^-1^.s^-1^)	*k*_d_ = *k*_off_ (s^-1^)	*K*_D_ = *k*_d_/*k*_a_ (M)
eL42	eRF1	5.3 x 10^3^	1.99 x 10^-4^	3.75 x 10^-8^
Δ(GGQTK)	eRF1	1.56 x 10^2^	4.87 x 10^-4^	3.12 x 10^-6^
eL42-Q51A/ Κ53Α	eRF1	2.34 x 10^2^	3.79 x 10^-4^	1.62 x 10^-6^
eL42-Q51E	eRF1	1.31 x 10^2^	2.51 x 10^-4^	1.92 x 10^-6^
eL42-K53Q	eRF1	1.47 x 10^2^	1.88 x 10^-4^	1.28 x 10^-6^
eL42	EF-3	4.9 x 10^3^	7.38 x 10^-4^	1.51 x 10^-7^
Δ(GGQTK)	EF-3	0.23 x 10^2^	2.18 x 10^-4^	0.92 x 10^-5^
eL42	tRNA-Asp	1.5 x 10^6^	2.3 x 10^-3^	1.5 x 10^-9^
Δ(GGQTK)	tRNA-Asp	5.89 x 10^4^	2.89 x 10^-4^	4.9 x 10^-9^
eL42	rRNA	4.56 x 10^6^	2.53 x 10^-4^	5.54 x 10^-11^
Δ(GGQTK)	rRNA	2.33 x 10^6^	1.18 x 10^-4^	5.05 x 10^-11^

## LIST OF ABBREVIATIONS

**The abbreviations used are:** CD, circular dichroism ; PTC, peptidyl transferase center ; tRNAox, periodate-oxidized tRNA, the 2’,3’-dialdehyde derivative of tRNA ; rp, ribosomal protein ; eL42, eukaryal or archaeal large subunit ribosomal protein L42 (formerly L42A or L42AB in yeast, L36a or L36a-like in human, or L44e in archaea); bL12 (formerly L7/L12), eubacterial large subunit ribosomal protein L12; *rpl42*^+^: the gene coding for the wild-type eL42; *Hma*, *Haloarcula marismortui*.
